# Intramyocardial dissecting hematoma after anterior myocardial infarction in a 36-year-old patient: a rare case report

**DOI:** 10.3389/fcvm.2026.1910250

**Published:** 2026-09-22

**Authors:** Xinhua Qin, Zeyu Chen, Hongjun Xiang

**Affiliations:** Department of Cardiology, Yongshun County People’s Hospital, Yongshun, Hunan, China

**Keywords:** cardiac magnetic resonance, heart failure, intramyocardial dissecting hematoma, ischemic cardiomyopathy, myocardial infarction, percutaneous coronary intervention

## Abstract

**Background:**

Intramyocardial dissecting hematoma (IDH) is a rare and potentially fatal form of myocardial rupture that usually occurs following acute myocardial infarction (AMI). Due to its low incidence and nonspecific clinical manifestations, IDH is frequently misdiagnosed as left ventricular thrombus or pseudoaneurysm. Multimodality imaging, particularly cardiac magnetic resonance (CMR), plays a pivotal role in establishing the diagnosis and guiding treatment strategies.

**Case presentation:**

A 36-year-old man with a long-term smoking history presented with progressive chest tightness and dyspnea for two weeks. Electrocardiography demonstrated pathological Q waves with persistent ST-segment elevation in leads V2–V6, suggestive of subacute anterior myocardial infarction. Laboratory investigations revealed elevated cardiac troponin T and markedly increased N-terminal pro-brain natriuretic peptide levels. Transthoracic echocardiography showed severe left ventricular systolic dysfunction with a left ventricular ejection fraction (LVEF) of 29% and a heterogeneous apical mass measuring 63 × 57 × 25 mm. Contrast-enhanced computed tomography and cardiac magnetic resonance imaging identified a large lesion extending from the interventricular septum to the apex, highly suggestive of intramyocardial dissecting hematoma. Coronary angiography revealed severe proximal stenosis and complete mid-segment occlusion of the left anterior descending artery (LAD). The patient underwent successful percutaneous coronary intervention with implantation of three drug-eluting stents, achieving TIMI grade 3 flow. Following guideline-directed medical therapy, the patient's symptoms improved significantly without hemodynamic deterioration or hematoma progression.

**Conclusion:**

This case highlights the importance of considering IDH in patients with subacute myocardial infarction presenting with left ventricular masses. Multimodality imaging, particularly CMR, is essential for differential diagnosis and therapeutic decision-making. Conservative management after successful revascularization may be feasible in hemodynamically stable patients with IDH.

## Introduction

Intramyocardial dissecting hematoma (IDH) is a rare and potentially fatal form of myocardial rupture that most commonly occurs following acute myocardial infarction (MI) (1). It is characterized by hemorrhagic dissection within the myocardial layers while preserving the integrity of both the endocardium and epicardium. Although the incidence of post-MI mechanical complications has decreased substantially in the reperfusion era, IDH remains associated with high morbidity and mortality because of its rarity and diagnostic challenges (2).

The clinical manifestations of IDH are highly variable, ranging from asymptomatic presentation to cardiogenic shock and sudden cardiac death. Due to overlapping imaging features, IDH is frequently misdiagnosed as left ventricular thrombus, pseudoaneurysm, or intracardiac tumors. Therefore, early recognition using multimodality imaging is crucial for accurate diagnosis and treatment planning. Cardiac magnetic resonance (CMR), with its superior tissue characterization capability, is considered the gold standard for diagnosing IDH (3, 4).

Currently, the optimal management of IDH remains controversial. While surgical intervention may be necessary in hemodynamically unstable patients, conservative treatment has been reported to achieve favorable outcomes in selected stable patients. Herein, we report a rare case of giant intramyocardial dissecting hematoma following subacute anterior myocardial infarction in a young patient. The diagnosis was established using multimodality imaging, including echocardiography, contrast-enhanced computed tomography, and CMR. The patient underwent successful revascularization of the culprit left anterior descending artery and achieved a favorable clinical outcome with conservative management.

## Case presentation

A 36-year-old male farmer was admitted to our hospital with progressive chest tightness and exertional dyspnea for two weeks. He denied chest pain, syncope, fever, or palpitations. His medical history was unremarkable for hypertension, diabetes mellitus, coronary artery disease, or previous cardiac surgery. However, he had a significant smoking history of approximately 20–30 cigarettes per day for more than 20 years.

On admission, his blood pressure was 108/89 mmHg, heart rate was 112 beats/min, respiratory rate was 21 breaths/min, and body temperature was 36.5 °C. Physical examination revealed coarse breath sounds with mild bibasal crackles. Cardiac auscultation demonstrated diminished heart sounds and a systolic murmur at the mitral area. No peripheral edema was observed.

Electrocardiography showed sinus tachycardia, pathological Q waves, persistent ST-segment elevation, and T-wave inversion in leads V2–V6, accompanied by left anterior fascicular block, suggesting subacute extensive anterior myocardial infarction (Figure 1).

**Figure 1:**
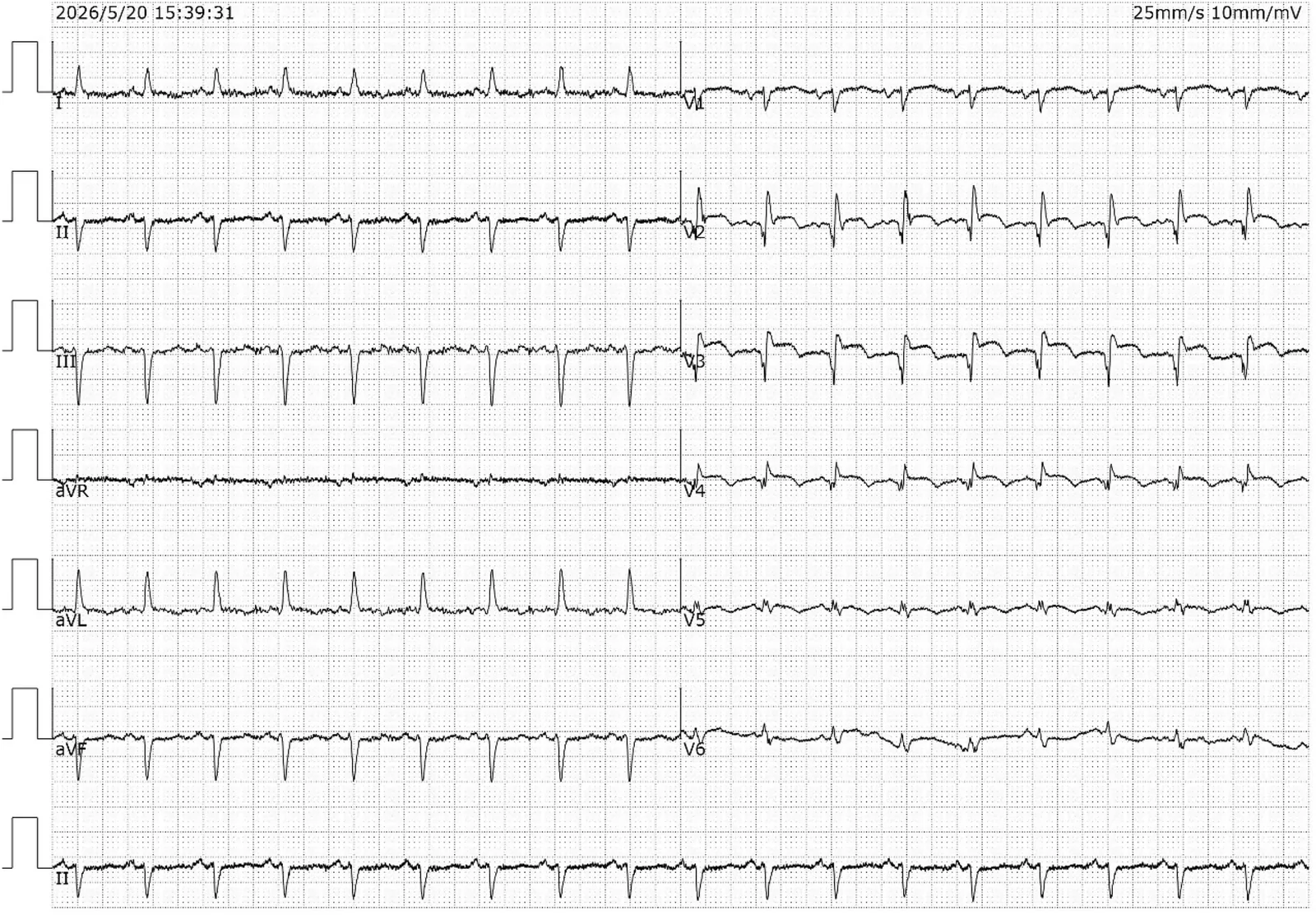
Admission electrocardiogram demonstrating subacute anterior myocardial infarction. The 12-lead electrocardiogram showed sinus tachycardia with pathological Q waves in leads V2–V4, persistent ST-segment elevation, and T-wave inversion in leads V2–V6, accompanied by left anterior fascicular block. The electrocardiographic findings suggested subacute extensive anterior myocardial infarction.

Laboratory testing revealed elevated cardiac biomarkers, including cardiac troponin T of 0.096 μg/L (reference range: 0.010–0.017 μg/L), markedly elevated N-terminal pro-brain natriuretic peptide (NT-proBNP) of 17,300 pg/mL, and D-dimer of 16,600 μg/L.

Chest computed tomography demonstrated cardiomegaly, right pleural effusion, and pericardial effusion. Transthoracic echocardiography revealed global left ventricular enlargement and severely impaired systolic function with an LVEF of 29%. A heterogeneous mass measuring approximately 63 × 57 × 25 mm was identified extending from the interventricular septum to the left ventricular apex (Figure 2). Differential diagnoses included intramyocardial hematoma, mural thrombus, or intracardiac mass. Moderate mitral regurgitation, severe tricuspid regurgitation, and mild pericardial effusion were also observed.

**Figure 2:**
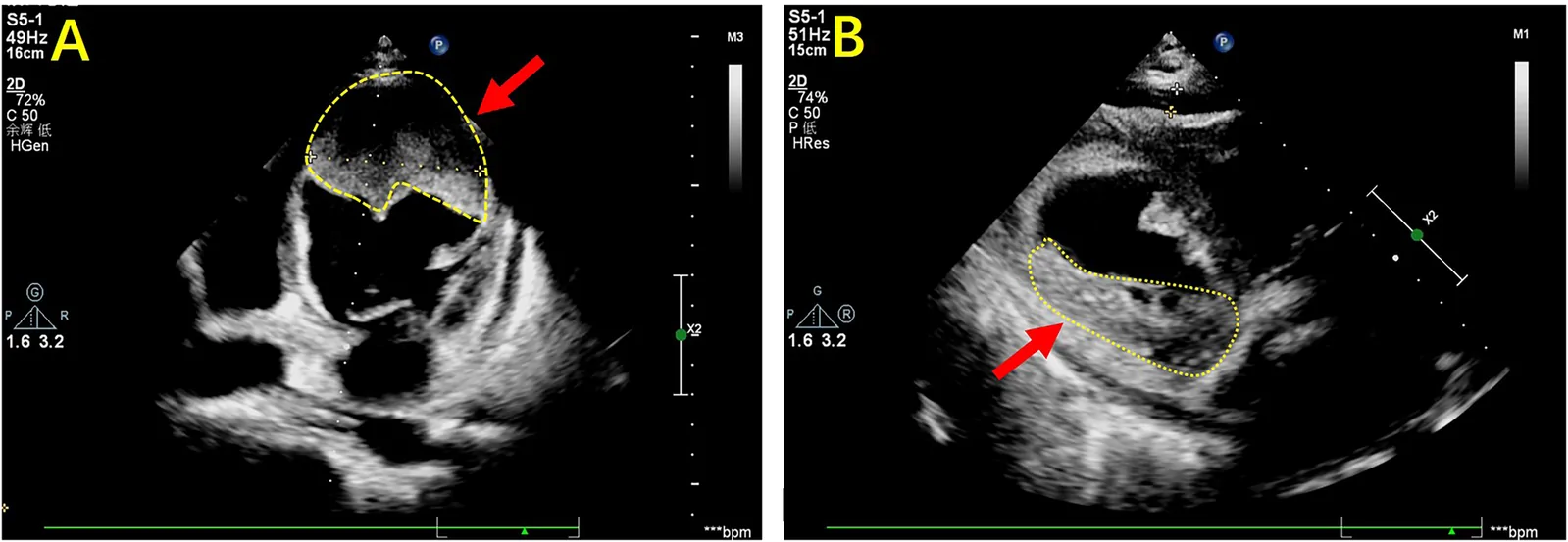
Transthoracic echocardiography demonstrating giant intramyocardial dissecting hematoma. **(A)** Apical four-chamber view showing a large heterogeneous cystic lesion involving the left ventricular apex (delineated by the yellow dashed line and indicated by the red arrow), measuring approximately 63 × 57 mm. The lesion was located within the myocardial wall and caused marked distortion of the apical geometry. **(B)** Parasternal long-axis view demonstrating a hypoechoic cavity extending from the interventricular septum toward the left ventricular apex (yellow dashed line and red arrow), consistent with intramyocardial dissection. The lesion was initially considered to represent left ventricular thrombus or other intracardiac masses. Combined with subsequent computed tomography and cardiac magnetic resonance findings, the diagnosis of giant intramyocardial dissecting hematoma was established.

Contrast-enhanced cardiac computed tomography demonstrated a low-density lesion involving the interventricular septum and apical region (Figure 3). Subsequent cardiac magnetic resonance imaging further characterized the lesion and strongly suggested a giant intramyocardial dissecting hematoma associated with transmural anterior myocardial infarction.

**Figure 3:**
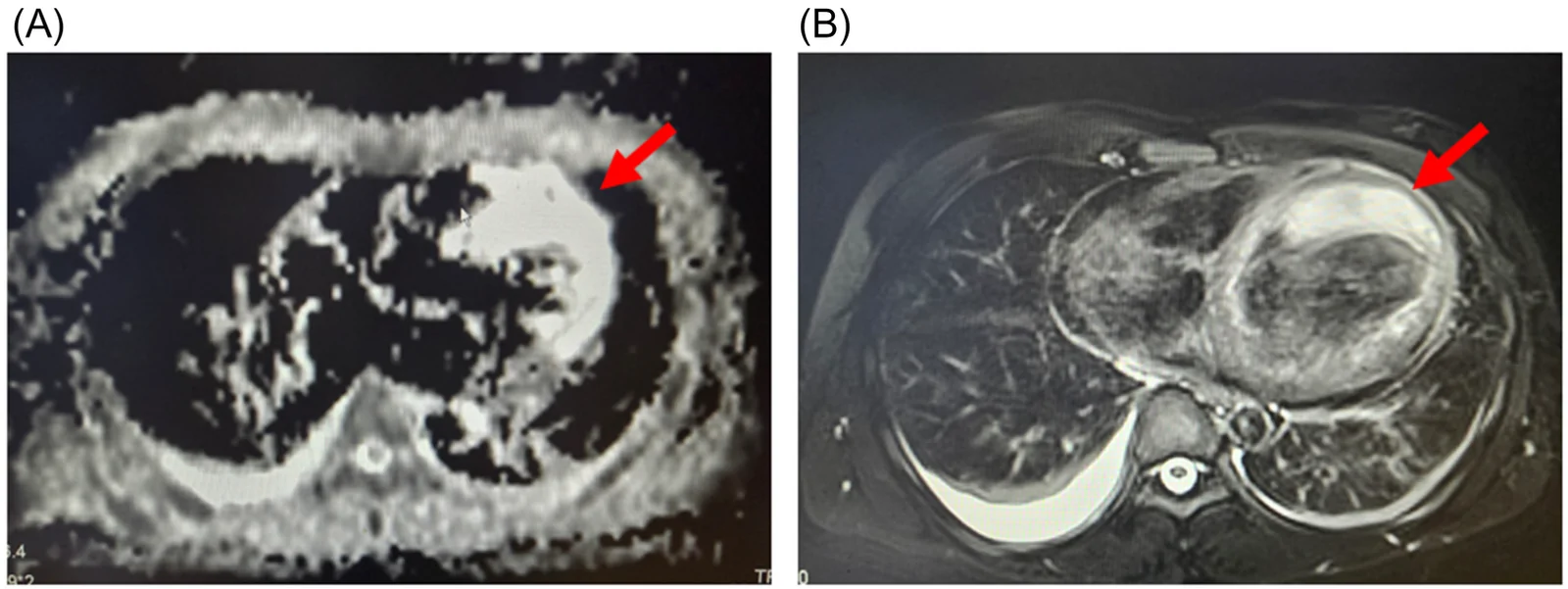
Cardiac magnetic resonance imaging confirming giant intramyocardial dissecting hematoma. **(A)** Cardiac magnetic resonance imaging demonstrated a large intramyocardial lesion involving the interventricular septum and left ventricular apex (red arrow). The lesion exhibited heterogeneous signal intensity and was confined within the myocardial wall. **(B)** Short-axis view showing a giant cystic lesion at the apical region (red arrow), surrounded by infarcted myocardium. CMR demonstrated a large intramyocardial cystic lesion at the interventricular septum and left ventricular apex, with surrounding akinesia. The lesion showed heterogeneous hyperintensity on T1- and T2-weighted imaging, consistent with subacute hemorrhage. Early gadolinium enhancement (EGE) showed no contrast entry into the lesion, excluding pseudoaneurysm. Late gadolinium enhancement (LGE) revealed transmural hyperenhancement of the adjacent myocardium, while the hematoma itself remained hypointense, with no pericardial leakage. These findings—contained intramyocardial lesion, mixed T1/T2 signal, absent early enhancement, and peripheral LGE—are diagnostic of intramyocardial dissecting hematoma, distinguishing it from left ventricular thrombus (homogeneous, no myocardial LGE) and pseudoaneurysm (contrast entry on EGE).

Coronary angiography revealed severe diffuse stenosis of the proximal left anterior descending artery, with approximately 95% luminal narrowing, and complete occlusion of the mid-LAD with TIMI grade 0 flow. Collateral circulation from the left circumflex artery to the distal LAD was present. The patient underwent successful percutaneous coronary intervention with implantation of three drug-eluting stents, restoring TIMI grade 3 flow (Figure 4).

**Figure 4:**
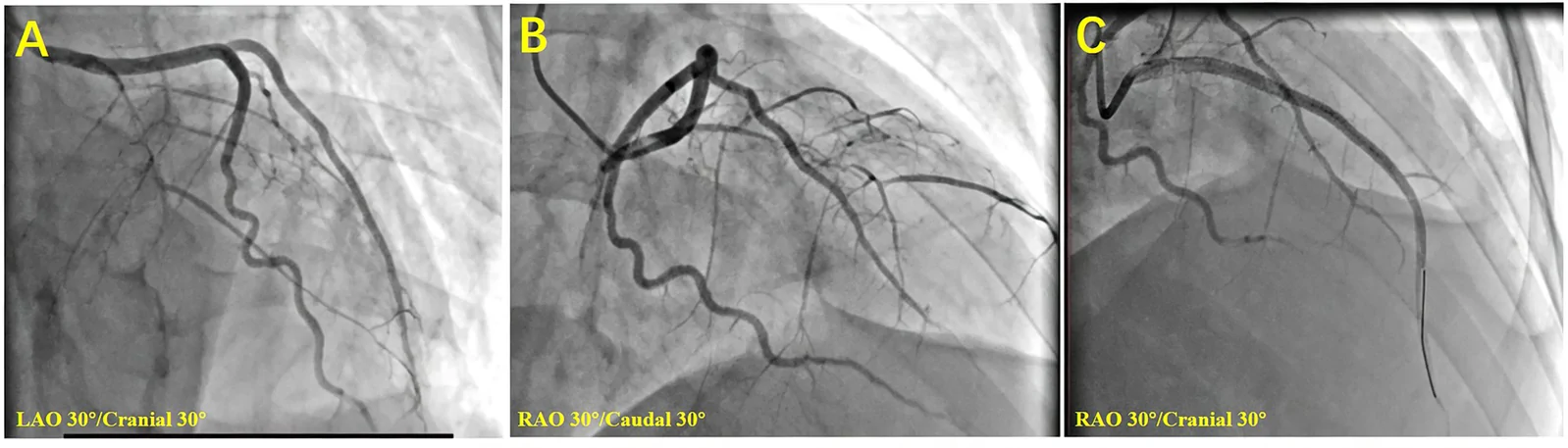
Coronary angiographic findings and percutaneous coronary intervention. **(A)** Coronary angiography revealed the mid-LAD with TIMI grade 0 flow in the left anterior oblique (LAO) 30°/cranial 30° projection. **(B)** Complete occlusion of the mid-LAD with TIMI grade 0 flow and collateral circulation from the left circumflex artery to the distal LAD were observed in the right anterior oblique (RAO) 30°/cranial 30° projection. **(C)** Successful percutaneous coronary intervention with implantation of drug-eluting stents restored TIMI grade 3 flow in the LAD in the right anterior oblique (RAO) 30°/cranial 30° projection.

Following revascularization, the patient received guideline-directed medical therapy, including dual antiplatelet therapy consisting of aspirin 100 mg once daily and clopidogrel 75 mg once daily, statins, beta-blockers, sodium-glucose cotransporter-2 inhibitors, and heart failure medications. At the time of discharge, repeat laboratory testing revealed a marked decrease in NT-proBNP to 4907 pg/ml. Follow-up transthoracic echocardiography demonstrated an improvement in LVEF from 29% to 35%, and a slight reduction in the size of the intramyocardial dissecting hematoma was observed, with no evidence of enlargement, pericardial effusion, or hemodynamic compromise. The patient was discharged in stable condition with regular outpatient follow-up scheduled.

## Discussion

Intramyocardial dissecting hematoma (IDH) is a rare subtype of myocardial rupture characterized by hemorrhagic dissection within the myocardial wall while preserving the integrity of the endocardium and epicardium. Most reported cases occur after acute myocardial infarction (MI), particularly involving the left anterior descending artery (LAD). Owing to its rarity and variable clinical manifestations, IDH remains a diagnostic and therapeutic challenge (5, 6).

Several features make the present case noteworthy. First, the patient was unusually young (36 years old) and heavy smoking was his only identified conventional cardiovascular risk factor. Second, he developed a giant IDH measuring 63 × 57 × 25 mm following subacute anterior MI complicated by severe left ventricular dysfunction. Third, multimodality imaging, including echocardiography, contrast-enhanced CT, and cardiac magnetic resonance (CMR), enabled accurate diagnosis. Finally, successful PCI combined with conservative treatment resulted in a favorable outcome. The decision to perform late percutaneous coronary intervention in our patient was based on the presence of progressive heart failure symptoms, extensive anterior ischaemia, and evidence of collateral-dependent viable myocardium. Although routine late revascularisation after MI remains controversial, it may be beneficial in selected patients with refractory symptoms or large areas of jeopardised myocardium (1). Given the considerable risk of further haematoma extension or myocardial rupture, we employed a meticulously gentle technique: low-pressure predilation with a 2.0-mm balloon, direct stenting to seal the dissection plane, avoidance of post-dilation, and careful anticoagulation. This cautious approach, coupled with the patient's stable haemodynamics, allowed successful restoration of TIMI 3 flow without procedural complications. Our experience suggests that in haemodynamically stable patients with IDH and viable myocardium, a strategy of conservative PCI with risk-mitigation measures can be safely performed, provided close clinical and imaging surveillance is maintained.

The pathogenesis of IDH is thought to involve extensive myocardial necrosis after transmural infarction, leading to rupture of intramyocardial vessels and hemorrhagic dissection along myocardial fibers. In the present case, complete mid-LAD occlusion and collateral circulation suggested prolonged ischemia before presentation, which likely contributed to the development of the hematoma (7).

The optimal management of IDH remains controversial. Surgical intervention is generally reserved for patients with hemodynamic instability or progressive hematoma enlargement. In contrast, conservative treatment may be appropriate for stable patients under close imaging surveillance. Despite severe left ventricular dysfunction, our patient remained hemodynamically stable after successful revascularization and showed significant clinical improvement with guideline-directed medical therapy (8, 9).

## Conclusion

This case highlights that IDH should be considered in patients with subacute MI presenting with persistent ventricular masses. Multimodality imaging, especially CMR, is essential for accurate diagnosis. Conservative management following successful revascularization may be a feasible strategy in selected hemodynamically stable patients with IDH.

## Data Availability

The original contributions presented in the study are included in the article/Supplementary Material, further inquiries can be directed to the corresponding author.
